# The File, as a Dental Instrument

**Published:** 1867-05

**Authors:** W. W. H. Thackston


### ARTICLE III.

### *The File, as a Dental Instrument.*

By W. W. H. Thackston, M.D., D.D.S.

Progress, is not always improvement. Change, as often travels downward, and backward, as upward and onward; and fashion, with its frailties and follies, plays not less fantastic tricks in the domain of science, than upon the broad surface of society. In this day of "radical" and startling change in the world without, and the world within, it may be profitable to get back to some of the old "landmarks," and take a "new departure."

The furor for vulcanite and coralite, for cheoplasty and chisels, for gouges and drills, for automatic and pneumatic pluggers, for rubber wedges and wooden wedges, for *mallets*, and may we not anticipate,—for sledges and tilt-hammers, for amalgams, "Hill's stopping," Oxide of Zinc, and "Woods' metal," for shreded gold and sponge gold, for the thousand and one *new* things that thrust themselves upon us at every turn, has appeared from our point of view, to cause many to overlook, or almost forget, a great deal that is *old* and *tried*, and which has been found *true* and *reliable*, and among other things, one of the oldest, simplest and yet when intelligently used, one of the most valuable and efficient instruments ever placed in the hands of the dentist.

The file, if not forgotten, seems only to be remembered to be decried and denounced in some "new light" Dental Associations, where prayers and politics, psalm-singing and soft-soldering-dental patents and "duplex elliptics" combine in one harmonious and delightful *melange;* and abundantly betoken the present status, and illustrate the present progress of American Dentistry.

No one attaches greater value, or places a higher estimate upon the operation of "filling teeth" when the work is done in a proper manner, and with a suitable material, than the writer of this article. "Tooth-filling" must ever constitute one of the leading features in dental surgery, and excellence in this individual operation, may well repay, and richly compensate a lofty ambition. And further, no one has a higher appreciation of the better class of operations usually denominated "mechanical" or "artificial," these are all indispensable, and invaluable in their several and proper places.

But with the foregoing acknowledgment and concessions, we maintain, that the (now almost obsolete) use of the file, is equally necessary to the best results desirable from correct dental practice—both in a prophylactic and remedial sense. A long, and somewhat critical observation in our own practice, and of the work of some of the Fathers of dental surgery (who have filled up the measure of their usefulness and honor, and passed away) as well as the practice of some of the most eminent who still survive, has abundantly satisfied us, that untold numbers of teeth are permitted to become the subjects of disease and decay, particularly upon their approximal surfaces, that might be protected, and preserved by the judicious use of the file. And more than this,—that *thousands* of teeth are sacrificed to a blind and over-weening confidence in the operation of "filling."

If compatible with the limits of this paper, or the object we have in view, we could present numerous and well authenticated instances in our own, and in the practice of more distinguished operators than we claim to be, of the permanent preservation of teeth by the file; which had been adjudged hopeless and incurable, so far as "filling" or any other operation could affect them, and which were *filed* as the only expedient, or experiment that could be applied. We have seen in the same denture, part of the teeth lost, under the best efforts of those most skilled in the operation of "filling;" and the remaining teeth, which had been abandoned as hopeless—a little mutilated, it is true, but sound and healthy, and satisfactorily performing all the functions peculiar to these organs.

We do not propose now to enter into any description of the *file*, its modifications of shape, its qualities of temper, cut or finish; nor do we design any observations upon the indications for its employment, or the best and most judicious methods of using this instrument, these subjects will all be more completely and clearly treated at another time, and in another place.

Our object in pening this article, is simply in our own plain way, to call back the attention of dentists, to an old, invaluable, but almost ignored or forgotten instrument; which used with judgment and skill has, and will continue to confer on humanity a blessed boon ; and upon dental science, some of its proudest trophies, and most brilliant triumphs. We wish to direct in some degree, the attention of the profession, from utter absorption in the more *doubtful* and *questionable* features, methods and materials of modern dentistry ; and induce practitioners, to consider well, if they have not already lost a vast amount of valuable substance in the eager chase after shadows, if they are not throwing aside old and better friends, than they are likely to make from the ephemera of the present day. We are not inimical to *true* progress and improvement, we are not wedded to old things, *because* they are old ; but experience has taught us to repose very little confidence in, and attach very little value to what *is simply new.*

We may say in conclusion, that the opinions and reflections we have submitted, are not wholly peculiar, they are shared, we are persuaded by some of the abler members of the profession, and we anticipate ere long, an endorsement and a more full and clear presentation of every point touched, by one of the most distinguished of the Alumni of the Baltimore College.

