# Errata

**Published:** 1867-07

**Authors:** 


*Errata.*—In the article on "The File as a Dental Instrument" in the May No. of the Journal, in the *first* sentence of the *third* paragraph the verb "to be" before the word "remembered" is superfluous.

In the second sentence of the same paragraph instead of the hyphen connecting the words "soldering—dental," there should be a comma.

In the *third* line of the *fifth* paragraph read the word "derivable" for "desirable." In the *seventh* line of the *eight* paragraph read "divert" instead of "direct."

